# Antagonistic potential of Trichoderma as a biocontrol agent against *Sclerotinia asari*

**DOI:** 10.3389/fmicb.2022.997050

**Published:** 2022-10-03

**Authors:** Zhiqing Wang, Ziqing Wang, Baohui Lu, Xingzhou Quan, Guangyuan Zhao, Ze Zhang, Wanliang Liu, Yixin Tian

**Affiliations:** ^1^College of Chinese Medicinal Materials, Jilin Agricultural University, Changchun, Jilin, China; ^2^College of Plant Protection, Jilin Agricultural University, Changchun, Jilin, China

**Keywords:** Trichoderma, *Sclerotinia asari*, biocontrol agent, antioxidant activity, metabolites, antifungal activity

## Abstract

In the present study, the inhibitory potential of 14 Trichoderma strains (isolated from Asarum rhizosphere) was investigated against *Sclerotinia asari* using the plate dilution method. The activity of antioxidant enzymes viz; catalase (CAT), peroxidase (POD), superoxide dismutase (SOD), and malondialdehyde (MDA) in *S. asari* treated with the two Trichoderma strains was also evaluated. Untargeted metabolomic analysis by using LC/MS analysis was carried out to determine differential metabolites in *T. hamatum* (A26) and *T. koningiopsis* (B30) groups. Moreover, transcriptome analysis of *S. asari* during the inhibition of *S. asari* by B30, and A26 compared with the control (CK) was performed. Results indicated that inhibition rates of *T. koningiopsis* B30, and *T. hamatum* A26 were highest compared to other strains. Similarly, non-volatile metabolites extracted from the B30 strain showed a 100% inhibition of *S. asari*. The activity of CAT, SOD, and POD decreased after treatment with A26 and B30 strains while increasing MDA content of *S. asari*. Antifungal activity of differential metabolites like abamectin, eplerenone, behenic acid, lauric acid, josamycin, erythromycin, and minocycline exhibited the highest inhibition of *S. asari*. Transcriptome analysis showed that differentially expressed genes were involved in many metabolic pathways which subsequently contributed toward antifungal activity of Trichoderma. These findings suggested that both Trichoderma strains (B30 and A26) could be effectively used as biocontrol agents against Sclerotinia disease of Asarum.

## Introduction

Plant diseases are one of the major factors that contribute directly to the depletion of agricultural natural resources (Benítez et al., 2004). These also contribute to 10–20% annual losses in global food production leading to a financial deficit of billions of dollars coupled with an adverse impact on food supply (Manczinger et al., 2002). Among plant pathogens, fungi are the most virulent pathogens among the soil-borne infections that cause significant losses. Out of many fungal infections in plants, necrotrophic fungi *Sclerotinia* spp. are among the most virulent pathogenic fungi causing infection in a wide range of crops and plants (Bolton et al., 2006). *Sclerotinia* spp. spend around 90% of their entire cycle as sclerotia in the soil (Kim and Knudsen, 2008). Sclerotia germinate during specific periods of the year, based on the fungus’s inherent potential and environmental factors, and create either mycelium or an apothecium to infect the host (Tyagi et al., 2020).

The *Sclerotinia* genus contains several species, each with a unique host organism on which it infects. *S. asari* is one of the members of the *Sclerotinia* fungal species that commonly infects Asarum (wild ginger) plants through its mycelium and causes Asarum sclerotiorum disease (Wang and Wu, 1983). *Asarum heterotropoides Var. Mandshuricum* is a popular perennial medicinal herb grown in the Liaoning Province of China and is commonly named Xixin (Huang et al., 2003). Essential oil isolated from Asarum *heterotropoides Var. Mandshuricum* possesses diverse therapeutic properties and is usually utilized as an analgesic, antibacterial, antifungal, and antitussive medicine in China (Perumalsamy et al., 2010). Previous studies have identified more than 82 compounds in the essential oil of Asarum with methyleugenol being the most prevalent compound (Zeng et al., 2004; Dan et al., 2010).

With the development of Asarum crops, Asarum sclerotiorum has emerged as a new concern. The disease was discovered in the Liaoning Province and Jilin Province in China (Wang and Wu, 1982). The *S. asari* has been found in both the underground and aerial sections of the affected plants. The disease causes rot in roots, seedlings, buds, stalks, and fruits eventually causing the entire plant to rot and die. Plant losses (that occur due to blighting) have been reported to be between 20 and 80% in cultivated gardens (Wang and Wu, 1984).

Various strategies including chemical and biological control methods are being employed to minimize the economic losses from plant pathogens like *S. Asari* infection. Chemical control methods have been effectively utilized to prevent plant diseases, but misuse has promoted the development of fungicide-resistant pathogens. Moreover, application of chemicals also destroys the ecological balance of soil microorganisms (Benítez et al., 2004; Kalia and Gosal, 2011). However, the development of genetic resistance against specific chemicals owing to genetic variations in populations also limits the potent effect of fungicides, whilst broad-spectrum fungicides also have adverse side effects on non-target species (Tjamos et al., 2013). Conventional chemical control approaches for many plant diseases may not always be cost-effective or efficient, and spraying and other chemical control methods might cause substantial health, safety, and environmental hazards (Monte, 2001). As a result, finding healthy, safe, and efficient biological control techniques has become an important task for the pathogen-free crops. Biological control is safe for humans and animals and also ecologically sustainable due to its diverse sources (Ling et al., 2021).

Biological treatment of plant diseases is usually practiced by using antagonists of phytopathogenic fungi, and 90% of such treatments have utilized various strains of Trichoderma fungus (Benítez et al., 2004). Majority of Trichoderma strains do not have a sexual lifecycle but rather generate just asexual spores. Moreover, several Trichoderma species are anamorphically associated with Hypocrea, and their Internal Transcribed Spacer (ITS) sequences have confirmed their phylogenetic closeness (Monte, 2001). The genus Trichoderma works as a biocontrol agent because of its ability to combat infections. Many genes of Trichoderma with potential antifungal effects have been isolated, cloned, and examined to determine their mechanism and function for various activities such as cell wall disintegration, hyphal development, and antagonist activity against phytopathogens (Sharma et al., 2011).

Trichoderma spp. isolated from seed and soil have shown distinct antifungal effects against *Rhizoctonia solani*, which is a pathogenic fungus that causes root rot and reduces bean yield (Mayo-Prieto et al., 2020). Moreover, volatile metabolites released from Trichoderma spp. have shown to inhibit the growth of soil-borne phytopathogens. Hydrolases such as chitinase and glucanase are among many of the metabolites that are assumed to be directly associated with mycoparasitism. To breakdown the cell walls of phytopathogen, the antagonistic Trichoderma synthesizes extracellular hydrolases which directly attack pathogens (Küçük and Kivanç, 2005). Recently, Ling et al. (2021) identified a strain of *Monosporascus ibericus* (AR-4-1) with the most potent antifungal effect against *S. asari* through the plate confrontation method from a collection of 31 strains having antagonistic effects on plant pests (Ling et al., 2021).

Previous studies have shown antagonistic potential of Trichoderma spp. against different phytopathogens by releasing metabolites and inhibiting their growth, however, the inhibitory potential of Trichoderma spp. against *S. asari* needs to be studied thoroughly because this phytopathogen is fatal for certain Asarum plants and can cause serious economic losses. Therefore, in the present study, the antifungal action of volatile and non-volatile compounds of two Trichoderma strains was tested against the *S. asari.* Metagenomic and metabolomic analysis was performed to investigate the antagonistic effect of volatile and non-volatile compounds of Trichoderma strains against the *S. asari* causing rot in *Asarum heterotropoides Var. Mandshuricum* plant. It was hypothesized that Trichoderma strains can be utilized as a biological control agent against the invading pathogen owing to its active metabolites to protect the Asarum crop.

## Materials and methods

### Isolation of Trichoderma strains and *Sclerotinia asari*

Trichoderma strains were isolated from the rhizosphere soil of healthy *Asarum heterotropoides var. mandshuricum* plants by the soil dilution method as described previously (Gil et al., 2009). Ten samples of the rhizosphere soil of healthy Asarum plants in Tonghua E125°34′, N41°78′) and Liuhe cities (E125°75′, N42°24′, Jilin Province were collected. Five-fold serial dilutions of every sample were made and 0.5 ml of the diluted sample containing Trichoderma spp. was poured on top of culture medium for culturing. Briefly, using the plate dilution method, 10 g of soil samples were added to 90 mL of sterile water, and mixed well. About 1 mL of this suspension was added into 9 mL of sterile water to make 10% soil suspension, and then diluted it with sterile water to 10^–3^ and spread it on the PDA medium. Mycelium at the edge of the colony was picked after cultivating for 3d with inversion culture at 28°C, and then inoculated it into a new PDA medium. Purified colonies were obtained after repeating above steps 5–6 times, and stored at 4°C for later use. Target fungus of *S. asari* was also isolated from the infected Asarum plants. The samples were carried to the lab and kept at 4°C until further processed.

### Molecular and morphological identification of Trichoderma isolates

The Trichoderma strains were inoculated into a PD liquid medium and placed in the culture plate in a shaking incubator for 5°days at 25°C. After that, the cells were centrifuged and freeze-dried at −80°C for 2–3°days before grinding into powder. The genomic DNA from Trichoderma culture was extracted as described previously (Möller et al., 1992). Two sets of double primers (nested primers) including one ITS primer ITS4 (5′-TCCTCCGCTTATTGATATGC-3′) and ITS1 (5′-TCCGTAGGTGAACCTGCGG-3′) was used to amplify a 600 bp fragment of ITS region of the ribosomal DNA (rDNA) gene. The PCR amplification conditions for this rection were: pre-denaturation at 94°C for 5 min; 35 cycles of denaturation at 94°C for 30 s, annealing at 48°C for 40 s, extension at 72°C for 1 min; and renaturation at 72°C for 10 min. The second set of primer was fRPB2-5F (5′-GAYGAYMGWGATCAYTTYGG-3′), fRPB2-7cR (5′-CCCATRGCTTGYTTRCCCAT-3′) that was used to amplify a 1,000 bp fragment of the second largest RNA polymerase subunit gene (RPB2 gene) and identify the target fungi (White et al., 1990). The PCR conditions for this amplification reaction were: pre-denaturation at 95°C for 5 min; denaturation at 95°C for 1 min, annealing at 55°C for 90 s, extension at 72°C for 90 s, 35 cycles; and final amplification at 72°C for 7 min. The obtained ITS sequences of the *Trichoderma* strains were compared to the related sequences in the GenBank database. Based on ITS-RPB2 double sequence alignment, phylogenetic tree of *Trichoderma* was constructed by using neighbor-joining method using MEGA11 software (Tamura et al., 2021). For morphological identification, *Trichoderma* strains were cultured on PDA and Oatmeal agar (OA) medium (30 g oat flakes, 13 g agar, per L of distilled water, pH 6.5) and characteristics of conidiophores were observed under high resolution compound microscope.

### Screening of Trichoderma strains for antagonistic activity

#### Determination of antagonistic effect of Trichoderma strains against *Sclerotinia sclerotiorum*

The activated *Sclerotinia sclerotiorum* cake (with a diameter of about 0.5 cm) was inoculated on the PDA medium. The fungal cake was put 2 cm away from the center of the medium and cultured at 25°C for 36°h. On the other side, a fresh Trichoderma strain cake, and the fungus cake was equidistant from the edge of the petri dish, and the control (without Trichoderma) was used as the blank. Each treatment was repeated three times. After 7°days, the diameter was measured by the cross method and the fungistatic rate was calculated using the following formula;


$$\mathrm{Fungistatic}\mathrm{rate}=\frac{\begin{array}{c} \mathrm{Diameter}\mathrm{of}\mathrm{control}\mathrm{colonies}-\text{Diameter}\;\mathrm{of}\;\text{treated colonies} \end{array}}{\mathrm{Diameter}\mathrm{of}\mathrm{control}\mathrm{colonies}}\times 100\%$$

#### Determination of the antagonistic effect of Trichoderma volatile substances on *Sclerotinia sclerotiorum*

The Trichoderma fungus cake was inoculated in the center of the PDA flat plate, and the bottom of another PDA flat plate was used to replace the top of the flat plate. After culturing at 25°C for 5°d, the diameter was measured by the cross method and the fungistatic rate was calculated using the above mentioned formula.

#### Determination of the antagonistic effect of non-volatile substances of Trichoderma on *Asarum sclerotiorum*

Sterile double cellophane on the PDA medium was spreaded, inoculated Trichoderma fungus cake in the center of the plate, and removed the cellophane with sterile tweezers when the mycelium grew to the edge of the cellophane. Then, the center of the plate was inoculated with *Asarum sclerotiorum* cake, and no Trichoderma was used in the control sample, and each treatment was repeated three times.

### Extraction of metabolites

After initial screening, non-volatile metabolites of two Trichoderma strains (*T. hamatum* A26 and *T. koningiopsis* B30) were extracted by ethyl acetate and water-saturated n-butyl alcohol. Fermentation broths of *T. hamatum* A26 and *T. koningiopsis* B30 were filtered and extracted with an equal volume of ethyl acetate. Both extracts were separately dried under vacuum at 40°C. After that, these extracts were dissolved in methanol, and the metabolite components of the extracts were identified by LC-MS analysis (Blaženović et al., 2018; Zeki et al., 2020).

### LC-MS conditions for differential metabolite analysis

A 20°μL of internal standard (L-2-chlorophenylalanine, 0.3 mg/mL; methanol configuration) was added to the samples (vortexed for 30 s, and sonicated for 3 min); and centrifuged for 10 min (at 13,000 rpm, 4°C). After that 150°μL of supernatant was removed with a syringe, filtered through a 0.22°μm organic phase pinhole filter, and then transferred to LC injection vials. It was then stored at −80°C until LC-MS analysis was performed.

Liquid-mass spectrometry system (AB SCIEX ExionLC 2.0+system, USA) containing ultra-high-performance liquid phase series (QE) high-resolution mass spectrometer was used to analyze the metabolome of two antagonist Trichoderma strains. Conditions for the liquid phase and mass spectrometry are given in Tables 1, 2.

**TABLE 1:** Liquid phase conditions for LC-MS analysis.

Parameters	Description
Column	ACQUITY UPLC HSS T3 (100 mm × 2.1 mm, 1.8°um)
Column temperature	45°C
Mobile phase	A-water (containing 0.1% formic acid) B-acetonitrile (containing 0.1% formic acid)
Flow rate	0.35°mL/min
Injection volume	2°μL

**TABLE 2:** Mass spectrometry conditions.

Parameter	Positive ions	Negative ion
Mass scan range	100–1,200	100–1,200
Resolution (full scan)	70,000	70,000
Resolution (HCD MS/MS scans)	17,500	17,500
Spray voltage (V)	3,800	−3,200
Sheath gas flow rate (Arb)	40	40
Aux gas flow rate (Arb)	10	10
Capillary temperature (°C)	350	350

### Determination of antioxidant activity

The activity of CAT, POD, SOD, and MDA enzymes of *S. asari* treated with Trichoderma strains was analyzed using the commercial kits following manufacturer’s instructions (Beyotime Biotechnology, Shanghai, China). Samples of treated *S. asari* were homogenized in 5 ml of 50 mM sodium phosphate buffer (pH 7.00). The homogenized sample was filtered before being centrifuged at 13,000 *g* for 20 min in a chilled centrifuge, and supernatant was used for further analysis. All procedures were carried out at 4°C according to recommended instructions for each analytical kit (Beyotime Biotechnology, Shanghai, China).

### Ribonucleic acid isolation, cDNA library preparation, and Illumina sequencing

For transcriptome analysis, firstly, the total RNA of 12 samples (four from each treatments A26, B30, and control group) was isolated according to the manufacturer’s instructions using the Qiagen RNeasy Plant Mini Kit (QIAGEN, Germany). Using a spectrophotometer (NanoDrop ND-1000; Thermo Fisher Scientific, Waltham, MA, USA), the quantity and quality of the RNA samples were determined. The TruSeq^®^ RNA Sample Preparation Guide from Illumina^1^ was used to create the fragment libraries. The Standard Cluster Development kit from Illumina was then used to load these libraries into the cluster generation station for single-molecule bridge multiplication. The Illumina Genome Analyzer I (GAI) was used to sequence the slide containing amplified clusters for single reads through using Illumina 36 cycle Sequencing Kit version 1. The Illumina Hi-Seq high-throughput sequencing platform sequences the cDNA library using Sequencing By Synthesis (SBS) technology, and the image data acquired using the high-throughput sequencer is transformed into a huge number of high-quality Data, known as raw data, by CASAVA base recognition (Caporaso et al., 2012).

### Transcriptome assembly and analysis

Raw sequencing reads with a quality score of less than 20 (≥ Q20) were eliminated using the cutadapt tool (Martin, 2011) and filtered to get clean reads. To screen clean reads, the FastQC v0.11.7 visualization program was utilized based on several parameters including, per-sequence quality score, GC content, error distribution, K-mer content, and sequence length distribution. Trinity was utilized in the project to join the clean reads. The contigs were clustered using the TGICL and Phrap programs to further combine the sequences and avoid any duplication at 90% sequence homology (Shentu et al., 2014). The Trinity Perl script was used to generate unigenes. The CD-HIT was used to integrate and cluster A26, B30, and CK clustered assemblies to build a A26_CK and B30_CK combined assembly. From these merged assemblies, unigenes were recovered. All samples were mapped against A26_CK and B30_CK unigenes with standard settings using the BWA-MEM tool (version 0.7.5). The final transcripts were utilized for downstream analysis.

### Gene functional annotation and differential analysis

The assembled reads were used to measure gene expression, and the transcripts were accurately determined by using Cufflinks program. The quantification of each gene was measured in the form of fragments per kilobase of exon per million mapped reads (FPKM) by using RSEM tool (Li and Dewey, 2011). Differential analysis of all possible samples’ combination was carried out by utilizing DESeq 2 (V 1.6.3) by using default parameters like *p*-values and log2FC. The unigene sequence was compared with GO, NT, NR, Swiss-Prot, KO, and KOG, databases using Blast2GO software V 3.0 (Conesa et al., 2005). After predicting the amino acid sequence of unigenes, the HMMER software (Finn et al., 2011), was used to search the Pfam database to obtain the annotation information of unigenes. Moreover, pathway analysis was performed on the predicted proteins using the Kyoto Encyclopedia of Genes and Genomes (KEGG). Using default parameters, the identified proteins were divided into five functional groups: biological processes, metabolic processes, Genetic Information Processing, cellular processes, and environmental Information Processing. The KEGG database was used to find metabolic pathways associated with important DEGs.

### Statistical analysis

All experimental results are presented as mean ± standard deviation. Analysis of variance (ANOVA) under completely randomized design was used to analyze the effect of treatments. Statistical significance was declared at *P* < 0.05. Statistical tools like R program were used for the statistical analysis of metabolome and transcriptomic data. Differential metabolites across samples were identified using VIP ≥ 1 and absolute Log2FC (fold change) ≥ 1.

## Results

### Morphological and molecular identification of Trichoderma isolates

**FIGURE 1:**
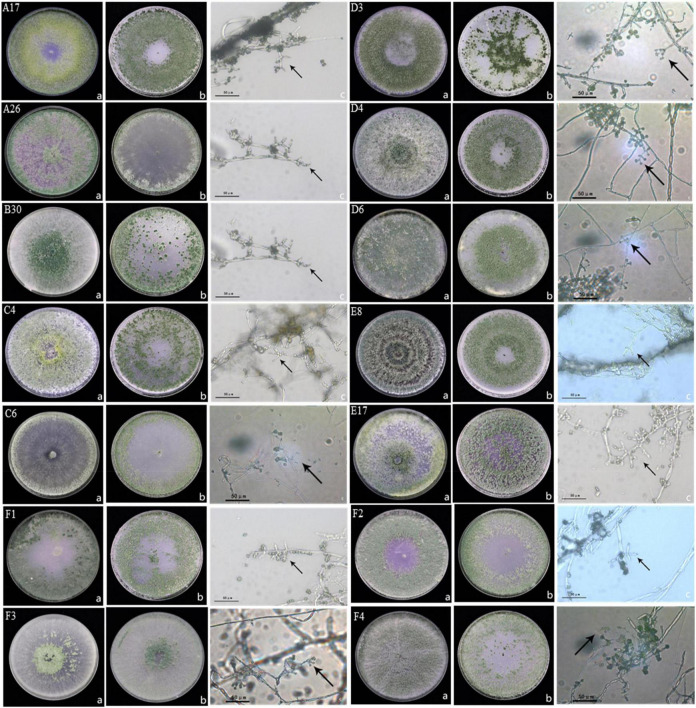
Morphology of *Trichoderma* strains **(A)** morphology of *Trichoderma* strains in PDA medium at 7d, **(B)** morphology of *Trichoderma* strains in OA medium at 7d, and **(C)** characteristics of conidiophore under a 40 × microscope; A17, C4, D4, D6, and E8 were classified as *Trichoderma harzianum*; B30, F1, and F2 were classified as *Trichoderma koningiopsis*; A26, C6, and F4 were classified as *Trichoderma hamatum*; E17 was classified as *Trichoderma atroviride*; D3 was classified as *Trichoderma brevicompactum*, F3 was classified as *Trichoderma tomentosum*.

Trichoderma isolates were identified by culturing *Trichoderma* strains on PDA and OA medium. Six species were identified including, *Trichoderma harzianum, Trichoderma hamatum, Trichoderma koningiopsis, Trichoderma atroviridae, Trichoderma brevicompactum*, and *Trichoderma tomentosum.* Morphology of *Trichoderma* strains on two different mediums and the conidiophores were visualized under high resolution microscope (Figure 1). *Trichoderma* strain B30 showed dense green conidiophores at the center of the plate under PDA medium while it was concentrated on the borders in OA medium. The A26 strain showed dispersed conidiophores at the borders in PDA (Figure 1).

**FIGURE 2:**
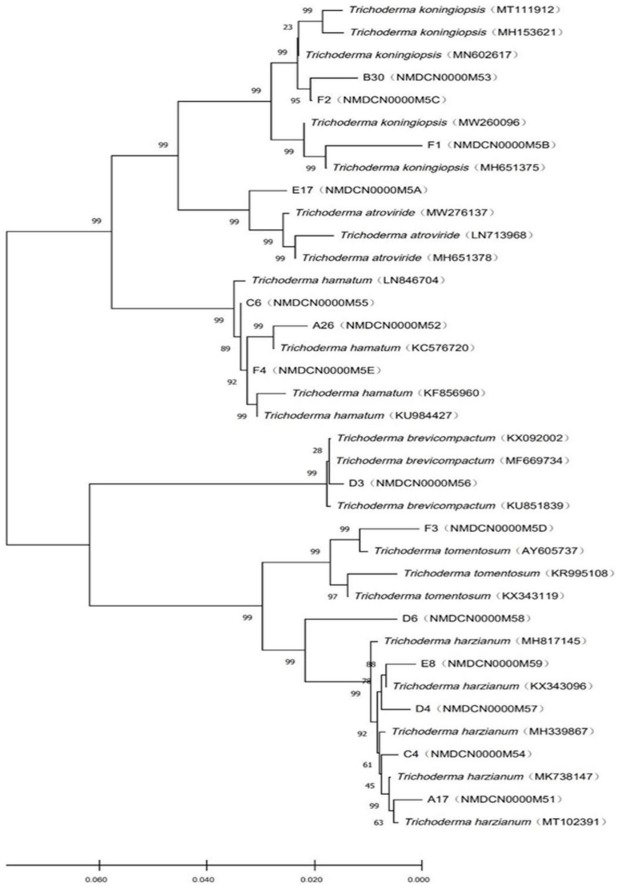
Phylogenetic tree of *Trichoderma* constructed based on the ITS-RPB2 double sequence alignment.

The objective of phylogenetic studies was to identify the isolates’ position and relationship with the known species of Trichoderma. In the phylogenetic tree, *T. koningiopsis, T. atroviridae*, and *T. hamatum* were belonged to the same clade and distinguished with bootstrap value of 99%. Similarly, *T. brevicompactum, T. tomentosum*, and *T. harzianum* were belonged to the same clade (Figure 2).

### Antagonistic activity of Trichoderma strains against *Sclerotinia asari*

**FIGURE 3:**
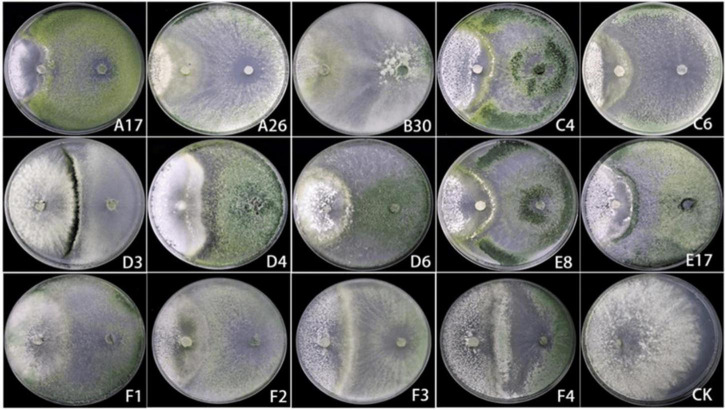
The confrontation effect of Trichoderma strains against *Sclerotinia asari*.

A total of 14 Trichoderma strains were isolated from the rhizosphere of *Asarum heterotropoides var. mandshuricum* plant. Trichoderma isolates showed rapid growth on PDA than pathogenic fungi *S. asari* under the same growth conditions. Results revealed that different isolates of Trichoderma inhibited the growth of phytopathogenic fungus *S. asari* to a varying extent (Table 3). However, highest inhibition rate was observed for two Trichoderma strains including A26 (92.77%), and B30 (91.32%). Whilst the lowest inhibition rate was shown by the D3 strain of *T. brevicompactum* (57.84%). The mycelial growth of the pathogenic fungi was suppressed substantially in the presence of an antagonist (Trichoderma strain) as compared to the control sample (Figure 3). All 14 Trichoderma strains exhibited different degrees of inhibition on the Asarum sclerotiorum. After 3°days, both fungi came in contact each other and *S. asari* fungus stopped growing. In the presence of strains A26, F1, and B30, the mycelial growth of the *S. asari* was reduced significantly (Figure 3 and Table 3).

**TABLE 3:** Inhibition rate of different Trichoderma strains against *Sclerotinia asari*.

Strains number	Inhibition rate (%)	Strains number	Inhibition rate (%)
A17	90.16 ± 0.02^a^	D6	79.34 ± 0.04^b^
A26	92.77 ± 0.00^a^	E8	89.26 ± 0.01^a^
B30	91.32 ± 0.01^a^	E17	88.06 ± 0.01^a^
C4	73.63 ± 0.01^bc^	F1	72.48 ± 0.01^cd^
C6	90.83 ± 0.01^a^	F2	76.86 ± 0.01^bc^
D3	57.84 ± 0.02^f^	F3	71.32 ± 0.00^cd^
D4	64.46 ± 0.05^e^	F4	67.35 ± 0.02^de^

Values with different lowercase letters indicate significant differences (*P* < 0.05).

### Effect of volatile and non-volatile metabolites of Trichoderma on the growth of *Sclerotinia asari*

Both volatile and non-volatile compounds of Trichoderma strains were extracted to evaluate their antifungal activity against the phytopathogen *S. asari* through in-vitro assay. All of the Trichoderma isolates synthesized harmful volatile compounds that had a substantial influence on the radial outgrowth of the *S. asari* (Table 4). The volatile metabolites of *T. hamatum* (C6) were more effective against *S. asari* as these compounds reduced mycelial growth up to 53.73%, followed by *T. koningiopsis* (B30) and *T. brevicompactum* (D3) strains with 45.77 and 37.61 percent inhibition, respectively. However, volatile metabolites from the *T. hamatum* (C4) strain showed the lowest inhibition rate (14.93%).

**TABLE 4:** Inhibition rate of volatile metabolites of Trichoderma strains against *Sclerotinia asari* ($\bar{\text{x}}$±s, *n* = 3).

Strain	Inhibition rate %	Strain	Inhibition rate %
A17	25.07 ± 0.03^efg^	D6	28.16 ± 0.02^ef^
A26	35.52 ± 0.01^cd^	E8	35.12 ± 0.04^cd^
B30	45.77 ± 0.06^b^	E17	29.35 ± 0.00^def^
C4	14.93 ± 0.03^h^	F1	22.99 ± 0.00^fg^
C6	53.73 ± 0.07^a^	F2	15.32 ± 0.02^h^
D3	37.61 ± 0.05^c^	F3	19.80 ± 0.05^gh^
D4	31.64 ± 0.02^cde^	F4	30.85 ± 0.02^de^

Values with different superscripts in same column differ significantly (*P* < 0.05).

Non-volatile compounds isolated from the *T. koningiopsis* (B30) strain showed 100% inhibition of the phytopathogen *S. asari*, followed by *T. hamatum* A26 (86.67%) and C6 (84.44%) strains (Table 5). The bacteriostatic rate of non-volatile substances of A17 and D3 strains was higher than 75%.

**TABLE 5:** Inhibition rate of non-volatile metabolites of Trichoderma strains against *Sclerotinia asari*.

Strain	Inhibition rate (%)	Strain	Inhibition rate (%)
A17	78.67 ± 0.01^bc^	D6	34.22 ± 0.03^f^
A26	86.67 ± 0.01^b^	E8	42.67 ± 0.11^ef^
B30	100.00 ± 0.00^a^	E17	40.44 ± 0.01^ef^
C4	45.78 ± 0.08^e^	F1	74.67 ± 0.04^c^
C6	84.44 ± 0.04^bc^	F2	64.44 ± 0.03^d^
D3	76.00 ± 0.01^c^	F3	58.22 ± 0.03^d^
D4	37.33 ± 0.04^ef^	F4	63.11 ± 0.03^d^

Values with different superscripts in same column differ significantly (*P* < 0.05).

### Metabolites of n-butyl alcohol extract

Based confrontation assay, two Trichoderma strains (A26 and B30) showing highest inhibition rate was selected for differential screening of metabolites through LC/MS analysis.

#### Screening of differential metabolites

**FIGURE 4:**
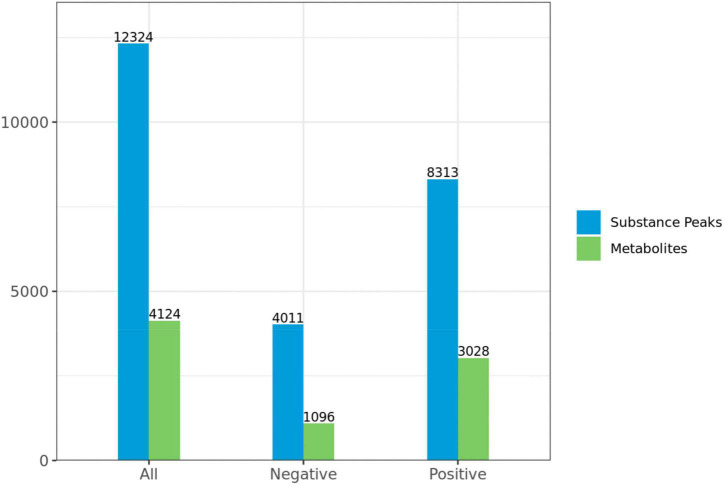
Statistics of substance peaks and metabolites.

**FIGURE 5:**
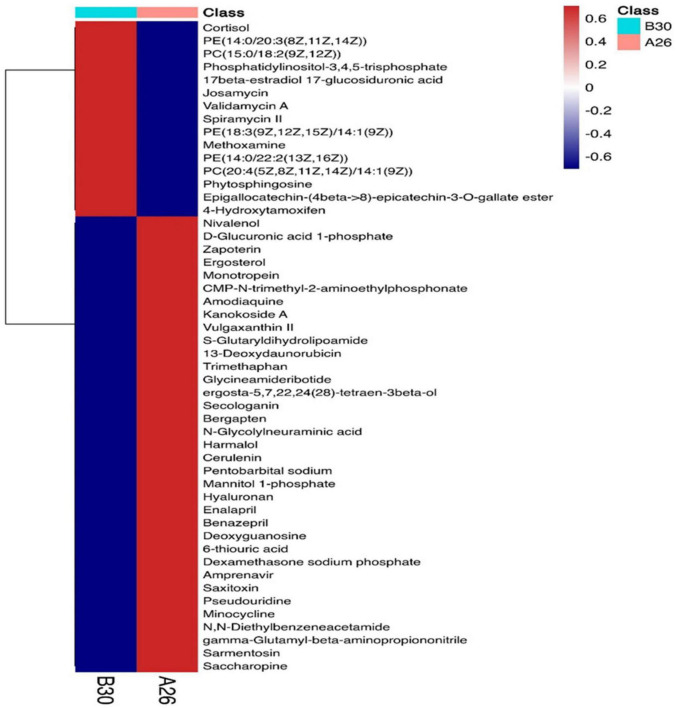
Heatmap cluster of top 50 differential antifungal metabolites.

**FIGURE 6:**
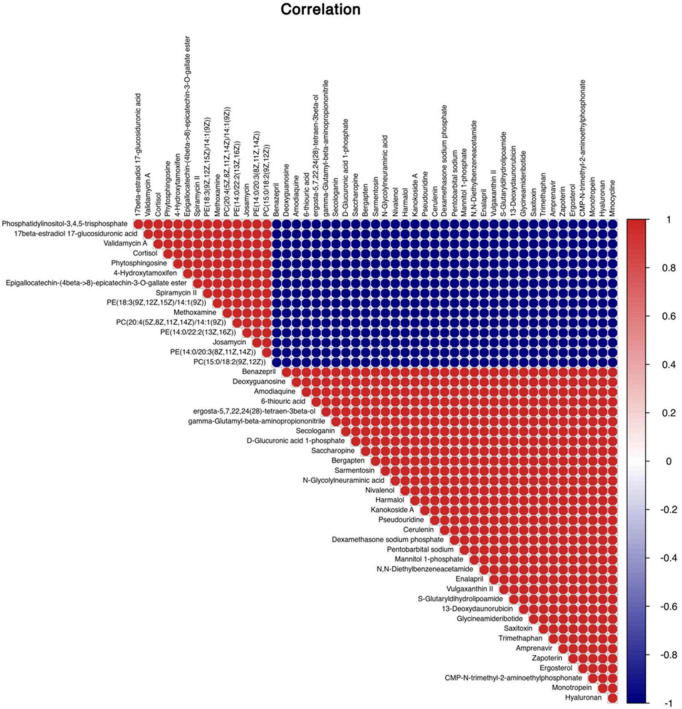
Correlation analysis between differential metabolites of A26 and B30 strains.

A total of 4,124 metabolites were identified through LC/MS analysis out of which 1,096 were negative ions metabolites 3,028 were positive ions (Figure 4). The positive and negative ion data were combined into a data matrix table containing all the information extracted from the original data and used for subsequent analysis. Out of total identified metabolites, 517 differential metabolites were screened having a valid ID. Out of all these identified anti-fungal substances, the top 50 differential metabolites from both strains are presented on the heatmap cluster diagram (Figure 5). Metabolites shown in red have a maximum score in each strain. Out of 50, about 15 metabolites were abundant in B30 strain including Cortisol, PE [14:0/22:2(13Z, 16Z)], Josamycin, Validamycin A, Methoxamine, Spiramycin, and phytosphingosine. In A26 strain, 35 metabolites were most abundant with the highest score including Nialenol, Zapoerin, Ergosterol, Monotropein, Minocyclin, Benazepril etc. Correlation cluster diagram was constructed to present the most abundant differential metabolites in both A26 and B30 strains (Figure 6). From the data, we have screened and narrowed down further to identify top 14 most effective and abundant antifungal substances from both strains, based on the relative abundance (peak value ≥ 10,000) against *S. asari* (Table 6).

**TABLE 6:** Chemical formula, retention time, classification information, and peak values of chemicals.

Metabolites	Retention time (min)	Super class	Formula	Mass error (ppm)	A26	B30
Eplerenone	10.2305	Lipids and lipid-like molecules	C_24_H_30_O_6_	−0.6162	5700417	5780019
Lauric acid	5.81235	Lipids and lipid-like molecules	C_12_H_24_O_2_	−0.44073	432089.8	724646.6
Behenic acid	10.83105	Lipids and lipid-like molecules	C_22_H_44_O_2_	−1.03304	1163034	636872.5
13Z,16Z-docosadienoic acid	15.69548	Lipids and lipid-like molecules	C_22_H_40_O_2_	−0.99472	223124.6	284071.3
Erythromycin C	13.55027	Organoheterocyclic compounds	C_36_H_65_NO_13_	1.637536	110266.8	219868
Josamycin	9.583817	Organic oxygen compounds	C_42_H_69_NO_15_	1.928782	0.000154	112371.3
Methyleugenol	4.6542	Benzenoids	C_11_H_14_O_2_	−0.45125	408.6141	38657.85
Epigallocatechin gallate	13.83662	Phenylpropanoids and polyketides	C_22_H_18_O_11_	−5.38426	440.3525	14620.66
Betaine	1.9765	Organic acids and derivatives	C_5_H_11_NO_2_	1.780473	35037.89	14607.93
2-Hydroxycinnamic acid	2.181733	Phenylpropanoids and polyketides	C_9_H_8_O_3_	−0.35413	165365.7	11617
Vanillin	4.073883	Benzenoids	C_8_H_8_O_3_	−0.48565	11015.62	3260.618
(2S)-Liquiritigenin	2.502067	Phenylpropanoids and polyketides	C_15_H_12_O_4_	−9.60528	52322.67	179.2478
Minocycline	5.250367	Phenylpropanoids and polyketides	C_23_H_27_N_3_O_7_	−2.73946	357687.4	0.000154
Avermectin	7.615217	Lipids and lipid-like molecules	C_49_H_74_O_14_	4.191199	44323.75	11993.04

#### Antifungal activity test of differential metabolites against *Sclerotinia asari*

**FIGURE 7:**
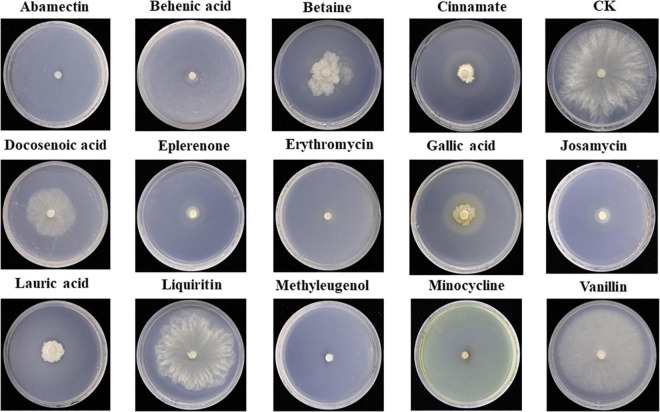
Antifungal activity of major differential metabolites against *Sclerotinia asari.*

Antifungal activity of the top 14 differential metabolites against pathogenic fungus *S. asari* was examined using the dilution method. All compounds exhibited antifungal activity against *S. asari* with varying degrees. Based on the clear zone diameter, Abamectin, Behenic acid, Eplerenone, Erythromycin, Josamycin, Methyleugenol, and Minocycline compounds showed higher antifungal activity (Figure 7).

#### Kyoto encyclopedia of genes and genomes classification and enrichment analysis

All differential metabolites were mapped through the KEGG (Kyoto Encyclopedia of Genes and Genomes) database. The KEGG classification and enrichment analysis showed that differential metabolites of Trichoderma are involved in many metabolic pathways like Tyrosine metabolism, Phenylalanine metabolism, pyrimidine metabolism, and galactose metabolism etc. Top 20 metabolic pathways were collected in which the amino acid metabolism (Tyrosine, phenylalanine, and Tryptophan metabolism) pathway possessed the highest number of metabolites, richness factor just less than 0.3, and *P*-value < 0.005 (Supplementary Table 7).

### Determination of malondialdehyde content and antioxidant activity

The *S. asari* treated with Trichoderma strains showed higher MDA content (Supplementary Table 1). The average MDA content of *S. asari* treated with replicates of A26, B30, and D6 strains of Trichoderma were 1.12 ± 0.02, 0.57 ± 0.10, and 0.52 ± 0.07 (μmol/g) respectively, were highest among other groups. The MDA content of *S. asari* after A26 treatment was 7.7 times greater than that of the control group. The MDA content of other treatment groups was significantly higher than the control group (*P* < 0.05).

After treatment with Trichoderma strains, the activity of antioxidative enzymes was decreased significantly in *S. asari.* The activity of SOD in *S. asari* was reduced significantly after treatment with Trichoderma strains D6 (15.49 ± 0.6°μ/g.min), D3 (21.89 ± 0.3 U/g.min), and C6 (25.92 ± 0.4 U/g.min) compared to control group (85.28 ± 3.1 U/g.min) (Supplementary Table 2).

Similarly, CAT activity was reduced significantly after treatment with Trichoderma strains E17, B30, and A26 (7.4 ± 1.3, 8.6 ± 0.4, and 16.2 ± 0.6 U/g.min, respectively), as compared to the control group (35.6 ± 0.7 U/g.min). The CAT activity of *S. asari* was reduced by 75.84% after treatment with B30 strain, which was significantly lower (*P* < 0.05) than other treatment groups (Supplementary Table 3). POD activity of *S. asari* treated with C6 was (8.33 U/g.min) decreased by 67.86% as compared to the control group (25.92 ± 3.20 U/g.min) (Supplementary Table 4).

### Illumina sequencing quality control and sequence assembly

#### Sequence data screening and assembly

The sequencing data obtained from strain B30 contains 97.33% proportion of clean reads, while A26 strains contain 98.39% of clean reads as compared to the control group CK1. The composition of filtered sequencing data of each sample is shown in Supplementary Figure 1. The error rate distribution plot of the sequencing data of each sample can be seen in Supplementary Figure 2. GC content along base reads obtained from B30 strain is 47.5% while it is 52.68% in A26 strain as compared to the CK1 GC content which is 46.09%. The GC content distribution map of each sample can be seen in the (Supplementary Figure 3).

The clean reads utilized in the following study are obtained after raw data filtering, sequencing error rate checking, and GC content distribution checking, and the data summary is provided in Supplementary Table 5.

After obtaining clean reads for projects with no reference genome, the clean reads must be spliced to produce reference sequences for later analysis. Trinity software generated a total 301,490,876 bp nucleotides with a mean contig length of 1697 bp, N50 of 2896 bp, and N90 of 690 bp. A total of 100,118 unigenes were generated with a mean length of 1,365 bp and N50 of 2,319 bp. The size distribution of these unigenes and transcripts length interval is shown in Supplementary Figure 4.

### Gene function annotation

**FIGURE 8:**
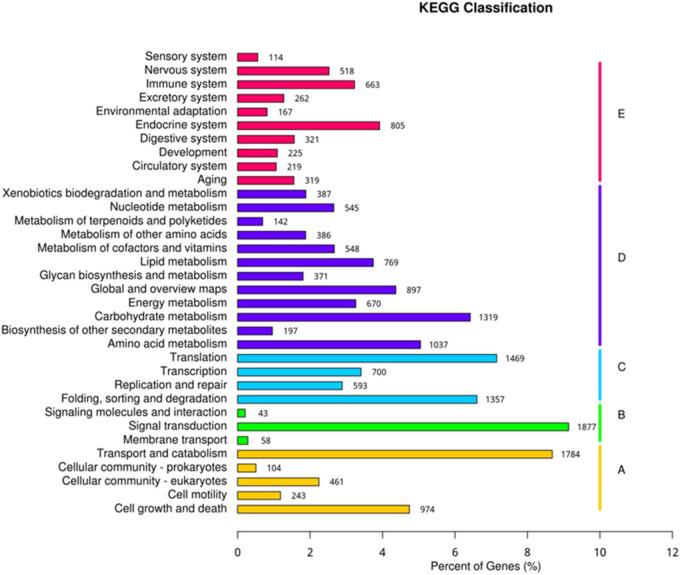
Kyoto encyclopedia of genes and genomes (KEGG) classification of genes expressed in different processes.

The Unigene sequences were compared with GO, NT, NR, Swiss-Prot, KO, and KOG, databases using BLAST, and after predicting the amino acid sequence of Unigene, the HMMER software was used to compare with Pfam database to obtain the annotation information of Unigene. Annotation of unigenes in seven databases was performed (Supplementary Figure 5) and the KEGG classification of genes involved in different processes was determined (Figure 8). Biological processes, metabolic processes, genetic information processing, environmental information processing, and cellular processes were the top processes involving highest number of genes. The present study identified 805 genes involved in the endocrine system, 1,319 genes in carbohydrate metabolism, 1,469 genes in the translation process, 1,877 genes in signal transduction, and 1,784 genes in the transport and catabolism processes (Figure 8).

### Genes quantification

#### Total gene count and distribution of all transcripts in each sample

Total reads of each sample were mapped to check the quantity of gene expression. The percentage of mapped genes from total reads was above 82% for every sample (Supplementary Table 6). For the calculation of the level of gene expressions in each sample, we determined expected Fragments Per Kilobase of transcript per Million mapped reads (FPKM) values which were substantially higher in both treatment groups as compared to the control group (Supplementary Figure 6).

### Analysis of differential gene expression

#### DEGs between different groups

**FIGURE 9:**
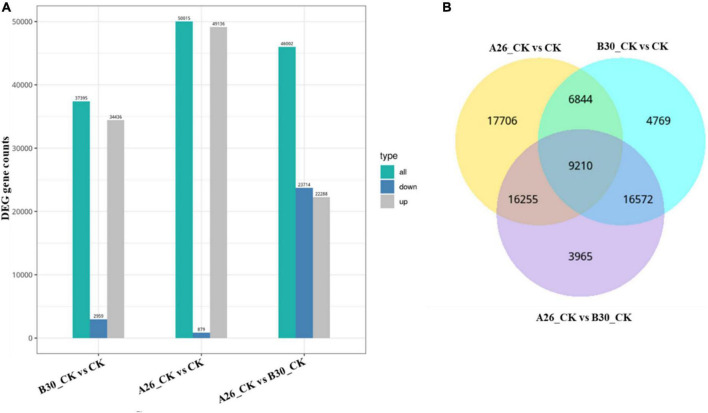
**(A)** Bar graph showing differential expressed genes between the different group, **(B)** VENN mapping shows common DEGs between different groups.

A total of 75,321 differentially expressed genes were annotated in all samples. In comparison with CK, a total of 37,395 DEGs of the B30_CK group were identified in which 34,436 genes were upregulated while 2,959 genes were downregulated. However, 22,288 DEGs were upregulated while 23,714 were downregulated between A26_CK and B30_CK treatment groups. Between A26_CK and CK groups there were only 879 genes downregulated while the remaining 49,136 were upregulated (Figure 9A).

#### Common and unique DEGs among treatment groups

Venn diagram analysis showed that A26_CKvsCK shared 6844 common DEGs with B30_CKvsCK, and 16255 genes with the A26_CKvsB30_CK group. While, 17706 DEGs were unique. Similarly, the A26_CKvsB30_CK group shared 16572 DEGs with B30_CKvsCK having 3965 DEGs unique. A total of 9,210 DEGs were common among all three groups (Figure 9B).

#### DEGs clustering and Kyoto encyclopedia of genes and genomes enrichment analysis

**FIGURE 10:**
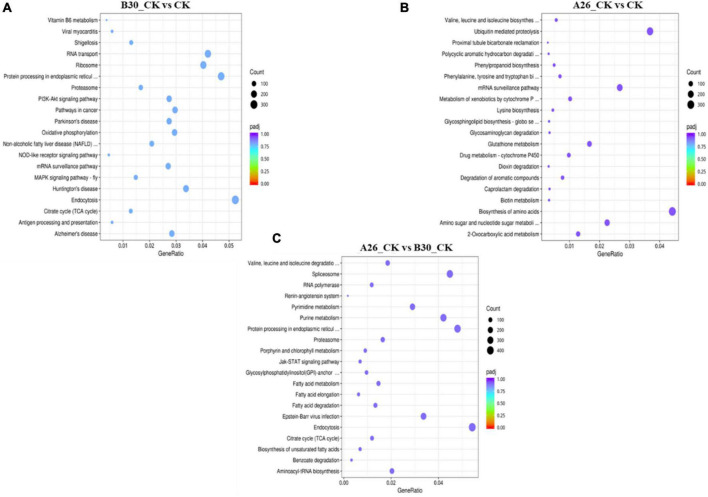
Kyoto encyclopedia of genes and genomes (KEGG) enrichment scatter plot of DEGs, **(A)** Top 20 enriched pathways of B30_CKvsCK **(B)** A26_CKvsCK **(C)** A26_CKvsB30_CK.

Heatmap cluster analysis of treatment groups exhibited significant differences in the gene expression (Supplementary Figure 7). Gene expression data from each sample were mapped to the KEGG pathway to determine involvement of DEGs in various classes of pathways. Using the R software package enrichR, we conducted pathway analysis and functional annotation for up and down-regulated genes. In B30_CK vs. CK group, a total of 37,395 DEGs were mapped to 347 KEGG pathways, out of which the top 20 enriched KEGG pathways are shown in Figure 10A. Similarly, the top 20 enriched pathways of DEGs from A26_CK vs. CK and B30_CK vs A26_CK are shown in Figures 10B,C, respectively. Results revealed that DEGs were highly enriched in several pathways like protein processing in the endoplasmic reticulum, Ubiquitin mediated proteolysis, Biosynthesis of amino acids, Endocytosis, Ribosome, and RNA transport (Supplementary Table 7).

## Discussion

In recent years, traditional Chinese medicine has gained much attention due to its benefits in preventing many diseases. Therefore, controlling the quality of Chinese herbal medicine is very crucial. *Asarum heterotropoides* Fr. Schmidt var. *mandshuricum* (Maxim.) Kitag is a valuable industrial and pharmaceutical plant that is used to cure a range of diseases (Huang et al., 2003). The roots or rhizome of the *Asarum* plant commonly known as Xixin in Chinese or Asari Radix *et* Rhizoma in the pharmaceutical industry is among the most prominent traditional Chinese herb (Jing et al., 2017). *S. asari* a pathogenic fungus infecting the Asarum plant is a growing concern. The biological treatment of phytopathogenic fungus *S. asari* is more efficient than chemical methods. For this purpose, Trichoderma strains have been used as a biocontrol agent because of their potential antifungal activity (Gajera et al., 2013).

In the present study, the plate confrontation method was used to check the antagonistic effect of two selected Trichoderma strains A26 (*T. hamatum*) and B30 (*T. koningiopsis*) against *S. asari.* Results indicated that both these strains exhibited the highest inhibition rates compared to the other strains. The morphological changes after the confrontation assay were also quite obvious. Previously, Yuan et al. (2017) reported the antimicrobial effect of *T. longibrachiatum* against *S. sclerotiorum* was highest compared to other pathogenic fungi including *Alternaria sclerotiorum* and *Rhizoctonia solani*. Moreover, *T. koningiopsis* has also shown a considerable inhibitory effect against *Sclerotinia*. Microscopic examination revealed that Trichoderma hyphae may grow alongside or penetrate and wrap around *S. sclerotiorum* hyphae, limiting their development and eventually causing *S. sclerotiorum* hyphae to disintegrate and damage (Kang et al., 2017). These findings clearly indicate the antagonistic activity and biocontrol mechanism of Trichoderma strains against phytopathogenic fungi.

Antifungal effects of metabolites produced by Trichoderma strains against *S. asari* were also investigated in the present study. Trichoderma produces a variety of secondary metabolites, including volatile and non-volatile compounds with potential antioxidant and antimicrobial activities (El-Hasan et al., 2007). The 6-pentyl-2H-pyran-2-one (6PP) is the only volatile metabolite, produced by different Trichoderma strains, which has been studied extensively with a major antagonistic activity against phytopathogens. The 6PP has shown inhibitory effects against *Fusarium oxysporum, Rhizoctonia solani*, and *Fusarium moniliforme* (Serrano-Carreón et al., 2004; El-Hasan et al., 2007; Vos et al., 2015). In the present study, lower inhibition rate of volatile substances produced by *T. hamatum* A26 and *T. koningiopsis* B30 indicated the limited role of volatile metabolites of both strains in antifungal activity against phytopathogens.

In the present study, inhibitory effect of non-volatile metabolites produced by *T. hamatum* A26 was > 85% and it reached up to 100% for the B30 strain, indicating that non-volatile metabolites of both strains possess a potent antagonistic effect. These findings are in agreement with earlier studies reporting the antagonistic activity of two Trichoderma strains (*T. harzianum* and *T. brevis)* against four pathogens causing rice seedling blight (Zhan et al., 2020). Non-volatile metabolites exhibited higher inhibition rate against *F. graminearum* as compared to the inhibitory effect of volatile metabolites. These findings are in agreement with previous studies that also evidently revealed a stronger inhibitory effect of non-volatile metabolites against phytopathogens than volatile metabolites (Tapwal et al., 2011; Khaledi and Taheri, 2016; Zhao et al., 2020).

In the current study, LC-MS analysis was carried out to screen differential metabolites from A26 and B30 strains. Analysis of antifungal activity revealed that seven out of 14 metabolites exhibited the highest antifungal activity against *S. asari.* These metabolites are well-known for their antibacterial and antifungal properties (Tan et al., 2021). Eplerenone has been identified as a novel antibacterial substance for drug repurposing against multi-drug resistant bacteria (Asghar et al., 2021). Moreover, Behenic acid has also shown bactericidal activity against *Agrobacterium tumefaciens* T-37 and *Ralstonia solanacearum* RS-2 (Xie et al., 2021). Similarly, antimicrobial and antifungal activities of other abundant metabolites, Josamycin, methyleugenol, erythromycin, and minocycline, identified in the present study have also been reported earlier (Anzaku et al., 2017; Dekhnich et al., 2018; Garcia-Jimenez et al., 2018; Keman and Soyer, 2019; Mohammed Ali Al-Harrasi et al., 2021).

In the present study, KEGG classification of differential metabolites showed that enriched metabolites mainly included; alkaloids and derivatives, lipids and lipid-like molecules, phenylpropanoids and polyketides, organic acids and derivatives, benzenoids, oxygen like nitrogen compounds, and organic oxygen compounds. These metabolites were involved in many metabolic pathways like tyrosine metabolism, phenylalanine metabolism, valine, leucine, and isoleucine biosynthesis, and pyrimidine metabolism. Metabolites present in tyrosine and phenylalanine metabolism pathways were upregulated indicating that these metabolites are necessary for the synthesis of many proteins involved in the development and growth. On the other hand, purine metabolism, lysine degradation, and taste transduction pathways were downregulated and only a few metabolites were identified in these pathways. Previous studies have shown that secondary metabolites synthesized by non-pathogenic fungal strains were converted into antifungal substances of several types, contributing to antifungal action (Strobel, 2003). Secondary metabolite synthesis (particularly phytoalexins) was found to be an essential plant defensive mechanism against plant diseases (Yuan et al., 2019). Antimicrobial phenolics such as lignins, cumarins, flavonoids, and isoflavonoids are formed from phenylpropanoid molecules. These metabolites are produced in response to microbial invasion and can stop the proliferation of infections (Harman et al., 2004; Huang et al., 2010).

In the present study, the activity of antioxidant enzymes like SOD, POD, CAT, and MDA was also determined to evaluate the effect of treatment (with Trichoderma strains) on antioxidant defense of *S. asari*. Lipid peroxidation refers to the interaction of reactive oxygen species (ROS) with lipids. MDA is a product of lipid peroxidation and a high level of MDA is an indicator of the damaged cell membrane (Morales and Munné-Bosch, 2019). SOD enzyme scavenges free oxygen radicals (O^–2^) into hydrogen peroxide H_2_O_2_, while, POD and CAT effectively remove H_2_O_2_ from the cell and maintain the active oxygen production in the cell (Inze, 2001; Schopfer et al., 2001; Liu et al., 2014). Libin et al. (2013) reported that the ethyl acetate extract of Trichoderma harzianum fermentation broth can reduce the content of soluble protein of *Phytophthora infestans*, while reducing the activity of bacterial protective enzymes (SOD, CAT, and POD), and increasing the MDA content in the bacterial body (Libin et al., 2013). Xun et al. (2013) observed that the ethyl acetate extract of strain T-43 fermentation broth has a significant effect on the main antioxidant enzymes (SOD, POD, CAT, and PPO) of pathogenic bacteria (Xun et al., 2013). This extract inhibited the growth of pathogenic bacteria by destroying the defense system of pathogenic bacteria. In the present study, MDA content was increased continuously upon treatment with antagonistic strains as compared to the control group. Similarly, the activities of antioxidant enzymes (SOD, POD, and CAT) in *S. asari* were significantly lower when treated with antagonist Trichoderma strains (A26 and B30) indicating their effectiveness against the *Sclerotinia* disease of *Asarum*.

In the present study, transcriptome analysis was also carried out to identify differential genes involved in biological pathways. Differentially expressed genes were identified according to the relative expression levels in different samples, and their functional annotation and enrichment analysis were performed (Wang et al., 2009). Results of DEGs analysis showed that, many upregulated DEGs were related to pathways like protein processing in the endoplasmic reticulum, biosynthesis of amino acids, amino sugar and nucleotide sugar metabolism, endocytosis, ribosome, and RNA transport pathways. Amino acid biosynthesis and protein processing in the endoplasmic reticulum are major pathways that are necessary for the synthesis and transport of proteins (Yang et al., 2021).

Previous studies have reported that antagonistic activity of Trichoderma spp. stems from the expression of certain genes which regulate the secretion of enzymes like chitinases, cellulases, glucanases, and xylanases which can degrade cell wall (Harman et al., 2004). Some of the Trichoderma genes encoding enzymes have been discovered to indirectly induce plant resistance (Bolar et al., 2000). Furthermore, the presence of Trichoderma has also shown to stimulate the expression of plant chitinases (Shoresh and Harman, 2010). Moreover, significantly higher enzymatic activity of β-1,3-glucanase and chitinases in postharvest anthracnose of chili pepper was observed after treatment with *T. koningiopsis*, than the control group in a previous study (Ruangwong et al., 2021).

Overall findings of the present study indicated the importance of enriched metabolic networks particularly protein synthesis and signal transduction pathways in the biocontrol mechanism of *T. koningiopsis* B30 and *T.* hamatum A26 strains against *S. asari*. Therefore, it might be a promising strategy by applying biological treatment using Trichoderma strains, *T. koningiopsis* B30, and *T.* hamatum A26 as antagonist against *Asarum sclerotiorum*. Our findings also provide practical insights about inhibition of fungal phytopathogens with non-volatile compounds and their potential use a green strategy to control crop pests.

## Conclusion

The present study concluded that treatment with Trichoderma substantially damaged the antioxidant defense of *S. asari* by decreasing the activities of its protective enzymes (POD, SOD, and CAT) while increasing MDA activity. Furthermore, non-volatile compounds of Trichoderma (Abamectin, Eplerenone, Behenic acid, Josamycin, Erythromycin, Methyleugenol, and Minocycline) showed promising antifungal activity for the inhibition of *S. asari.* Findings of the present study indicated that *T. Koningiopsis* B30, and *T. hamatum* A26 could be used as biocontrol agents for the treatment of *Asarum Sclerotiorum*. Moreover, our findings provided molecular insights about the metabolic pathways responsible for the production of antifungal metabolites which can effectively inhibit the growth of *S. asari* and save the plant from fungal diseases.

## Data availability statement

The datasets presented in this study can be found in NCBI SRA under the project number PRJNA820374 (https://www.ncbi.nlm.nih.gov/bioproject/?term=PRJNA820374).

## Author contributions

ZhW designed the experiment, methodology, conducted the experiment, analyzed the data, and wrote the manuscript. ZhW, XQ, and ZZ performed the experiment and analyzed the data. BL performed the methodology. GZ supervised and revised the draft. WL contributed to project administration. YT contributed to writing—review and editing. All authors have read and approved the final version of this manuscript.

## Footnotes

1
http://www.illumina.com
