# Supplementary material for: Antagonistic potential of Trichoderma as a biocontrol agent against Sclerotinia asari
Source: Front Microbiol. 2022 Oct 3;13:997050. doi: 10.3389/fmicb.2022.997050 (PMC9578005; doi:10.3389/fmicb.2022.997050)
Supplement: Supplementary file 1 [file Data_Sheet_1.docx]

**Figures S-1** Composition chart of raw sequencing data


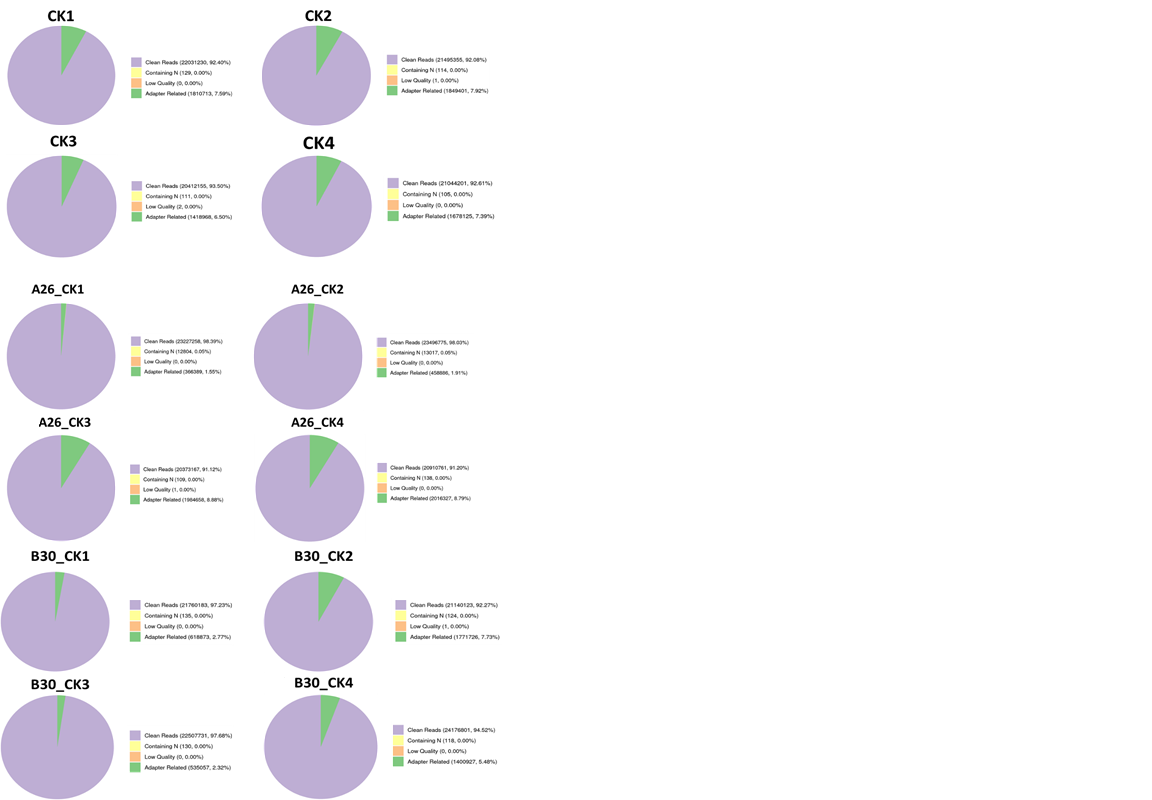


***Figures S-2*** Error rate distribution graph of


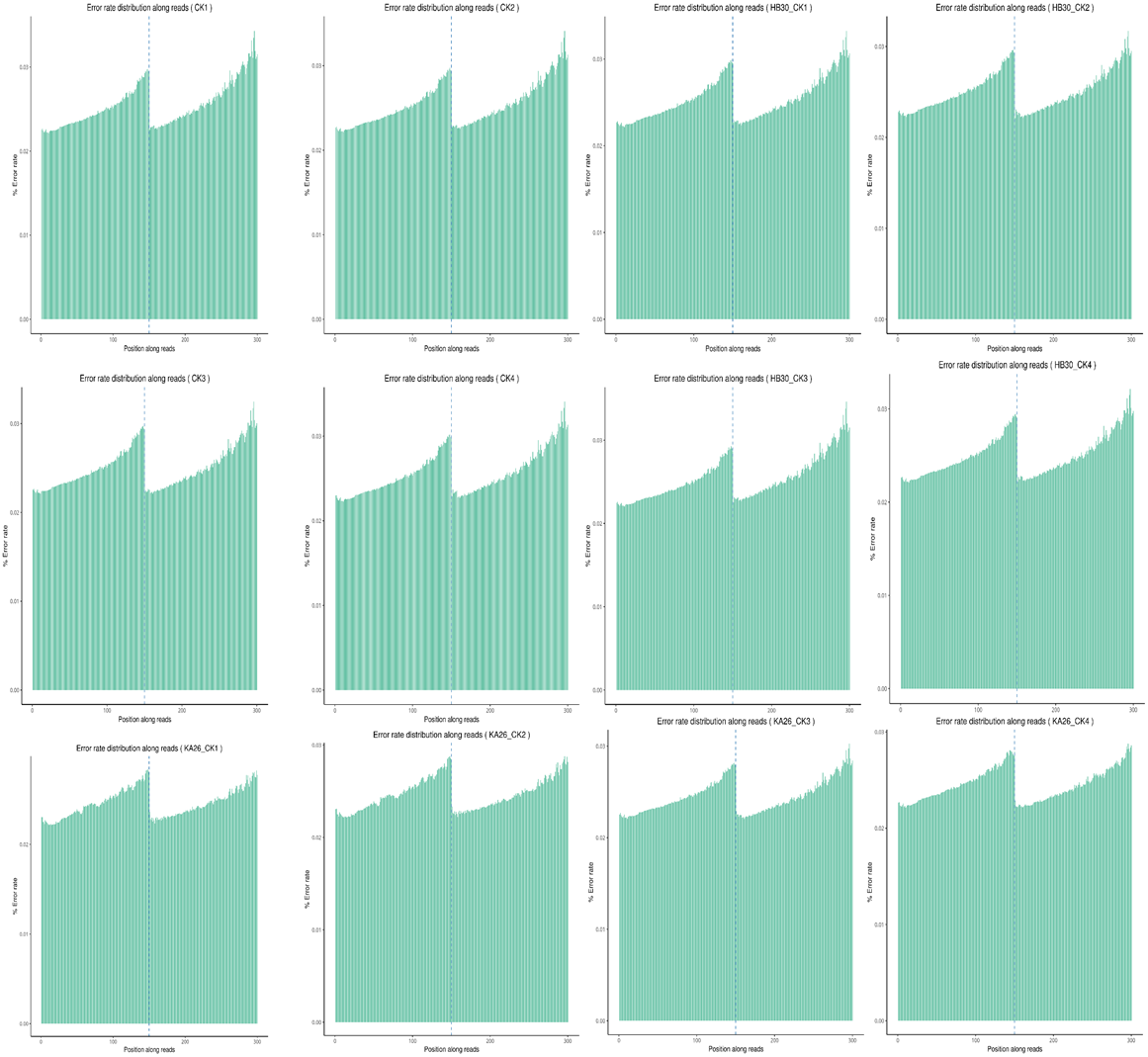


**Figures S-3** Bases (GC and AT) contents along reads of different samples


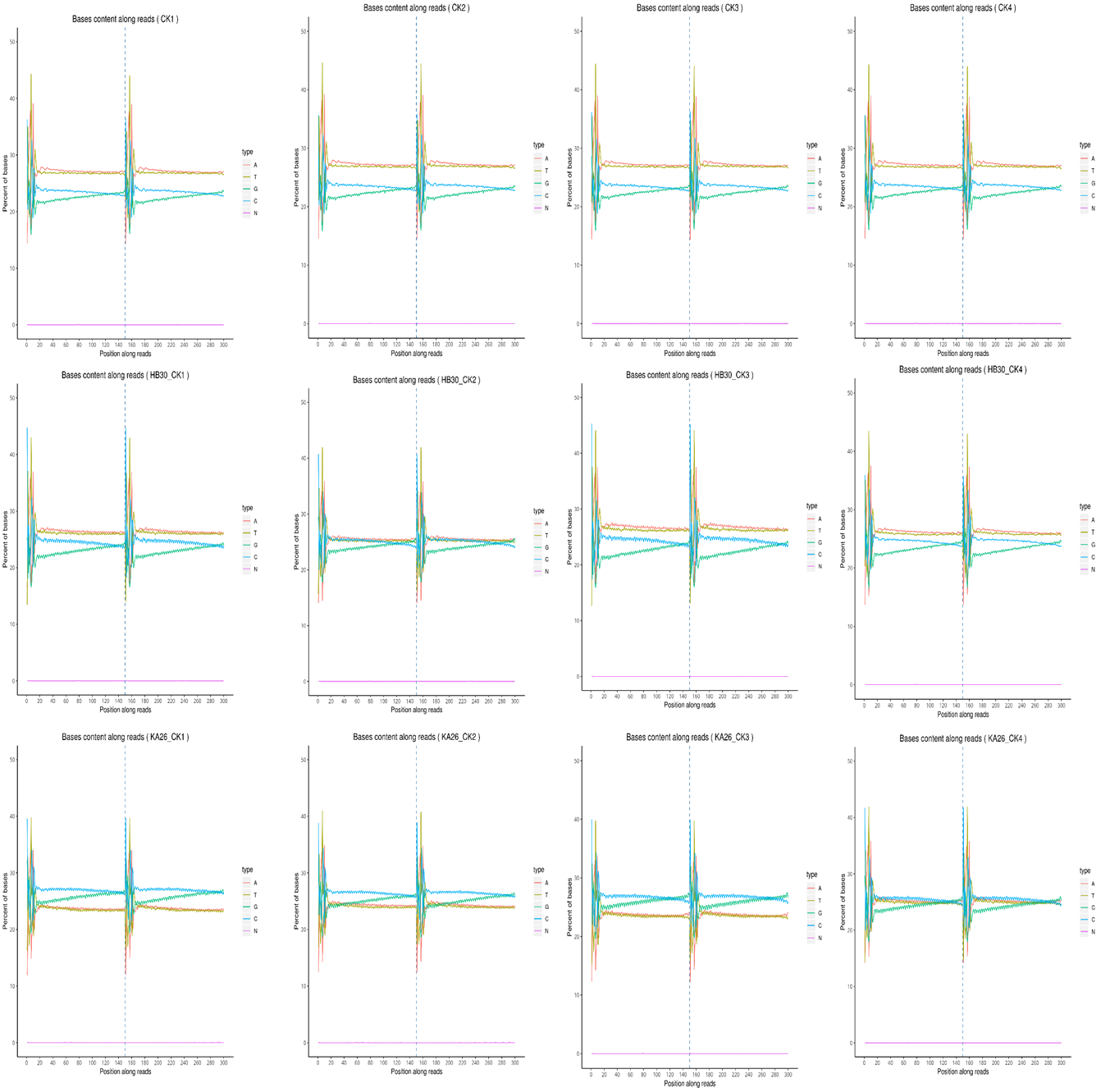


**Figure S-4** Sequence length distribution plot


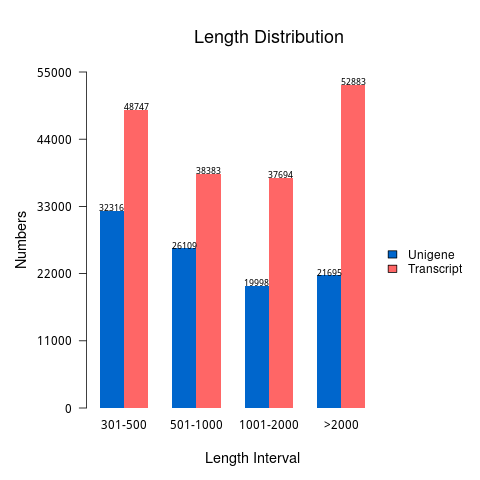


**Figure S-5** Annotation of unigenes in seven databases

**
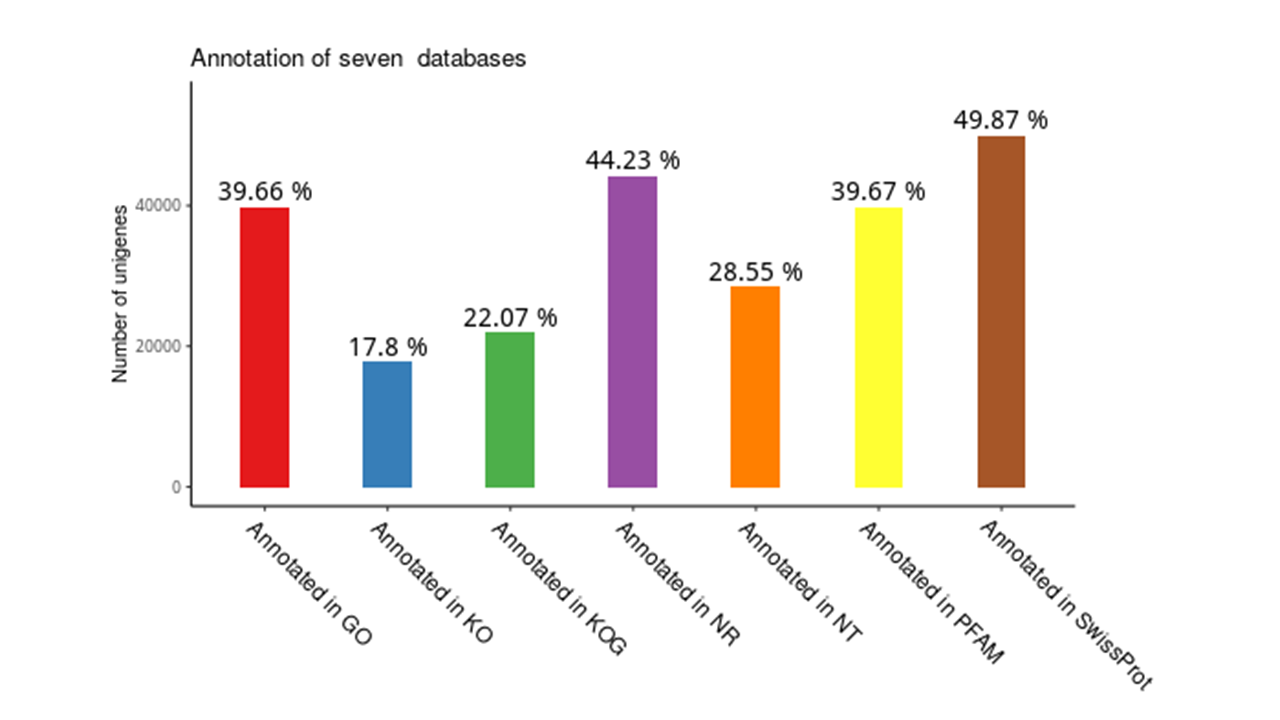
**

**Figure S-6** (A) FPKM distribution in treatment groups (B) FPKM density distribution across groups

**
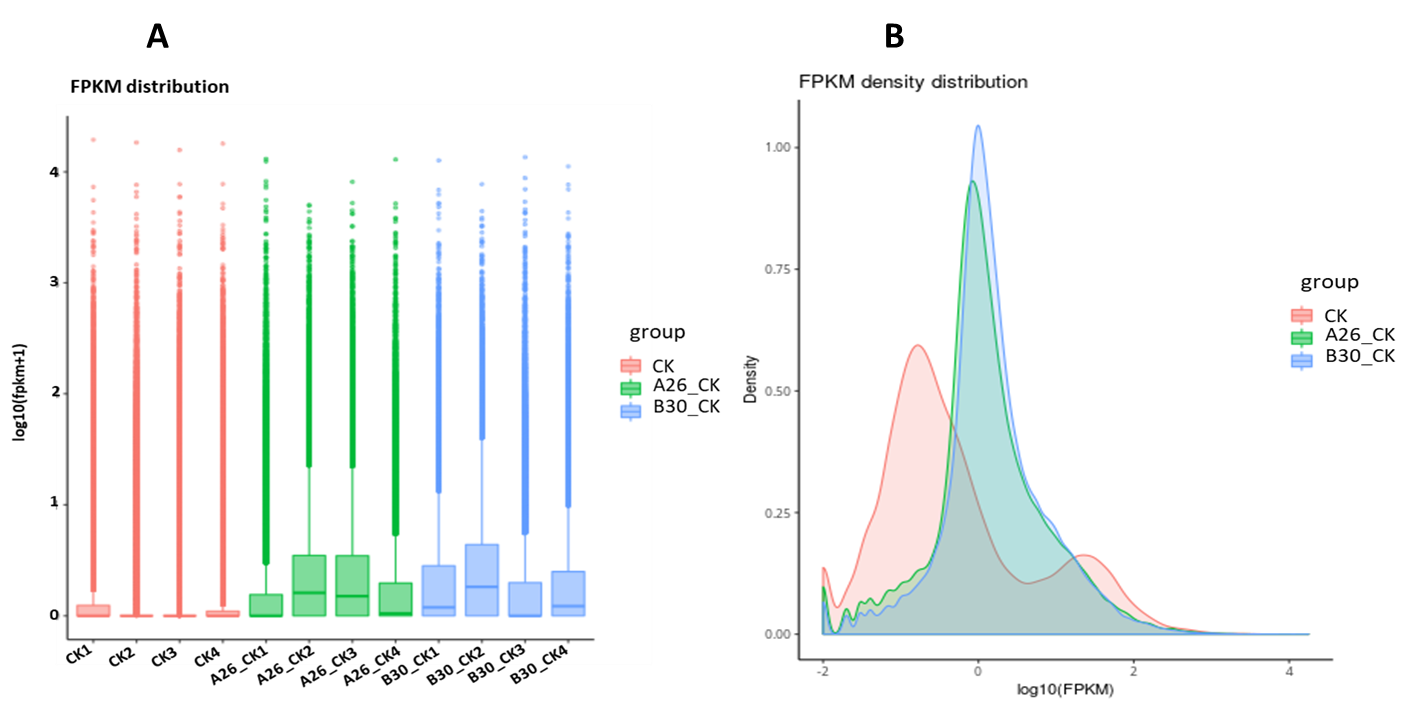
**

Figure S-7. Heatmap cluster analysis of DEGs in treatment and control groups

**
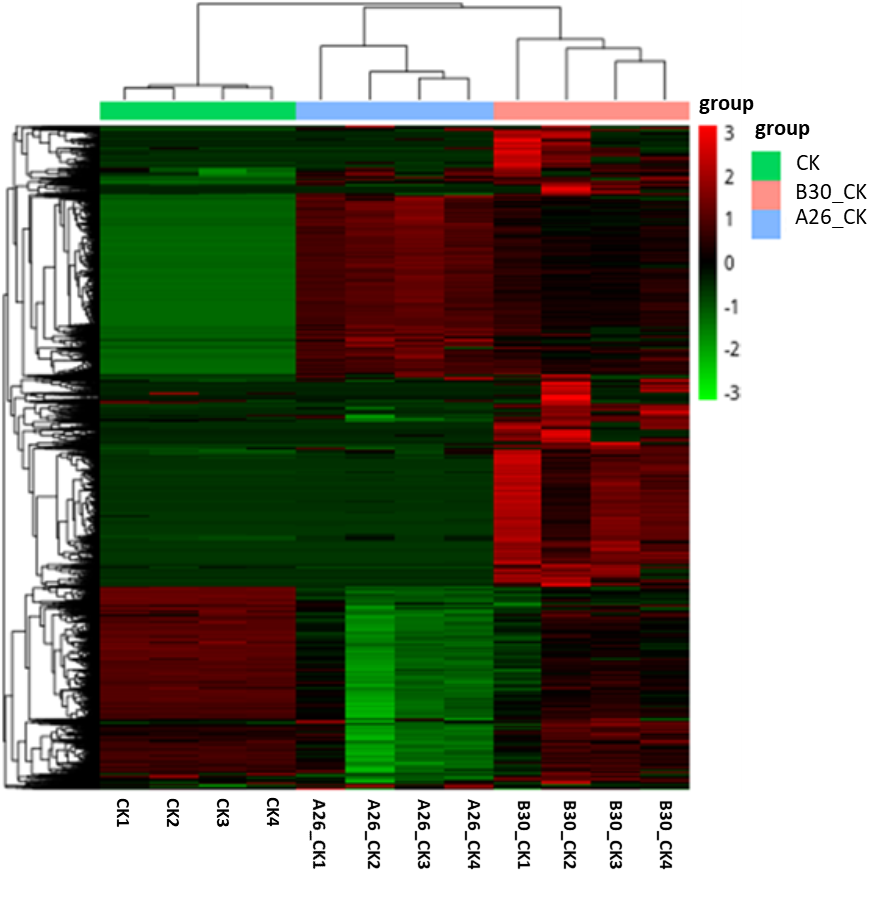
**

**Table S-1. Change of MDA content of *Sclerotinia asari* after treatment with *Trichoderma* strains**

| Strains | Strains replicate MDA content  (μmol/g) | | | Standard deviation | Mean |
| --- | --- | --- | --- | --- | --- |
| CK | 0.13 | 0.14 | 0.17 | 0.02 | 0.15 |
| A17 | 0.31 | 0.33 | 0.28 | 0.03 | 0.31 |
| A26 | 1.12 | 1.11 | 1.15 | 0.02 | 1.12 |
| B30 | 0.54 | 0.68 | 0.48 | 0.10 | 0.57 |
| C4 | 0.15 | 0.12 | 0.13 | 0.02 | 0.13 |
| C6 | 0.21 | 0.23 | 0.23 | 0.01 | 0.22 |
| D3 | 0.15 | 0.14 | 0.13 | 0.007 | 0.14 |
| D4 | 0.08 | 0.09 | 0.09 | 0.008 | 0.085 |
| D6 | 0.57 | 0.54 | 0.44 | 0.07 | 0.52 |
| E8 | 0.37 | 0.38 | 0.43 | 0.03 | 0.40 |
| E17 | 0.17 | 0.17 | 0.10 | 0.04 | 0.15 |
| F1 | 0.14 | 0.14 | 0.10 | 0.02 | 0.13 |
| F2 | 0.08 | 0.10 | 0.10 | 0.01 | 0.09 |
| F3 | 0.35 | 0.32 | 0.46 | 0.10 | 0.38 |
| F4 | 0.38 | 0.45 | 0.50 | 0.06 | 0.44 |

**Table S-2. Change of SOD activity of *S. asari* after treatment with Trichoderma strains**

| **Strains** | **Strains replicate SOD activity** | | | | **Standard deviation** | | **Mean** | |
| --- | --- | --- | --- | --- | --- | --- | --- | --- |
|  | **(U/(g·min)** | | | |  |  |  |  |
| **CK** | 88.29 | 85.59 | 81.98 | 3.16 | | 85.29 | |  |
| **A17** | 73.87 | 75.68 | 74.77 | 0.90 | | 74.77 | |  |
| **A26** | 47.03 | 46.49 | 47.03 | 0.31 | | 46.85 | |  |
| **B30** | 46.49 | 47.57 | 47.57 | 0.62 | | 47.21 | |  |
| **C4** | 46.49 | 45.95 | 45.95 | 0.31 | | 46.13 | |  |
| **C6** | 25.83 | 26.43 | 25.53 | 0.46 | | 25.93 | |  |
| **D3** | 21.89 | 22.16 | 21.62 | 0.27 | | 21.89 | |  |
| **D4** | 27.03 | 27.03 | 27.03 | 0.00 | | 27.03 | |  |
| **D6** | 14.86 | 15.95 | 15.68 | 0.56 | | 15.50 | |  |
| **E8** | 27.03 | 22.97 | 32.13 | 4.59 | | 27.38 | |  |
| **E17** | 47.57 | 46.49 | 47.57 | 0.62 | | 47.21 | |  |
| **F1** | 43.78 | 44.32 | 44.86 | 0.54 | | 44.32 | |  |
| **F2** | 23.24 | 24.86 | 28.11 | 2.48 | | 25.41 | |  |
| **F3** | 38.38 | 38.92 | 39.46 | 0.54 | | 38.92 | |  |
| **F4** | 56.22 | 57.30 | 45.41 | 6.58 | | 52.97 | |  |

**Table S-3. Change of CAT (Catalase) activity of *S. asari* after treatment with Trichoderma strains**

| **Strains** | **Strains replicate CAT activity** | | | | **Standard deviation** | | **Mean** | |
| --- | --- | --- | --- | --- | --- | --- | --- | --- |
|  | **(U/(g·min)** | | | |  |  |  |  |
| **CK** | 34.8 | 36 | 36 | 0.7 | | 35.6 | |  |
| **A17** | 25.2 | 24 | 24 | 0.7 | | 24.4 | |  |
| **A26** | 16.8 | 16.2 | 16.2 | 0.6 | | 16.2 | |  |
| **B30** | 8.4 | 9 | 8.4 | 0.3 | | 8.6 | |  |
| **C4** | 34.8 | 36 | 36 | 0.7 | | 35.6 | |  |
| **C6** | 27.6 | 28.8 | 25.2 | 1.8 | | 27.2 | |  |
| **D3** | 34.8 | 36 | 36 | 0.7 | | 35.6 | |  |
| **D4** | 25.8 | 28.2 | 27.4 | 1.4 | | 27.4 | |  |
| **D6** | 18 | 18.6 | 19.2 | 0.6 | | 18.6 | |  |
| **E8** | 25.2 | 27 | 24.6 | 1.2 | | 25.6 | |  |
| **E17** | 6 | 8 | 7.8 | 1.2 | | 7.4 | |  |
| **F1** | 30.6 | 33.6 | 33.6 | 1.7 | | 32.6 | |  |
| **F2** | 25.2 | 24 | 25.8 | 0.9 | | 25 | |  |
| **F3** | 34.8 | 34.2 | 32.4 | 1.2 | | 33.8 | |  |
| **F4** | 30.6 | 31.8 | 30 | 0.9 | | 30.8 | |  |

**Table S-4. Change of POD (Peroxidase dismutase) activity of *S. asari* after treatment with Trichoderma strains**

| **Strains** | **Strains replicate POD activity** | | | | **Standard deviation** | | **Mean** | |
| --- | --- | --- | --- | --- | --- | --- | --- | --- |
|  | **(U/(g·min)** | | | |  |  |  |  |
| **CK** | 27.8 | 27.8 | 22.2 | 3.2 | | 25.9 | |  |
| **A17** | 25 | 25 | 25 | 0 | | 25 | |  |
| **A26** | 16.7 | 12.5 | 12.5 | 2.4 | | 13.9 | |  |
| **B30** | 16.7 | 16.7 | 16.7 | 0 | | 16.7 | |  |
| **C4** | 15 | 15 | 20 | 2.9 | | 16.7 | |  |
| **C6** | 8.3 | 8.3 | 8.3 | 0 | | 8.3 | |  |
| **D3** | 20.8 | 16.7 | 15 | 3.0 | | 17.5 | |  |
| **D4** | 16.7 | 16.7 | 16.7 | 0 | | 16.7 | |  |
| **D6** | 13.3 | 16.7 | 12.5 | 2.2 | | 14.2 | |  |
| **E8** | 25 | 16.7 | 23.3 | 4.4 | | 21.7 | |  |
| **E17** | 18.8 | 14.3 | 11.1 | 3.8 | | 14.7 | |  |
| **F1** | 25 | 20 | 21.9 | 2.5 | | 22.3 | |  |
| **F2** | 12.5 | 15 | 16.7 | 2.1 | | 14.7 | |  |
| **F3** | 12.5 | 16.7 | 25 | 6.4 | | 18.1 | |  |
| **F4** | 20 | 16.7 | 11.1 | 4.5 | | 15.9 | |  |

**Table S-6.** Statistical table of sequencing output

| Sample | Library | Raw reads | Raw bases | Clean reads | Clean bases | Error rate | Q20 | Q30 | GC% |
| --- | --- | --- | --- | --- | --- | --- | --- | --- | --- |
| CK1 | FRAS210153715-6r | 23842072 | 7.2 | 22031230 | 6.6 | 0.03 | 97.93 | 93.72 | 46.09 |
| CK2 | FRAS210153715-7r | 23344871 | 7 | 21495355 | 6.4 | 0.03 | 97.96 | 93.8 | 45.89 |
| CK3 | FRAS210153715-8r | 21831236 | 6.5 | 20412155 | 6.1 | 0.02 | 98.11 | 94.16 | 45.96 |
| CK4 | FRAS210153715-9r | 22722431 | 6.8 | 21044201 | 6.3 | 0.03 | 97.88 | 93.6 | 46 |
| A26_CK1 | FRAS210153716-1b | 23606451 | 7.1 | 23227258 | 7 | 0.02 | 98.16 | 94.63 | 52.68 |
| A26_CK2 | FRAS210153716-2b | 23968678 | 7.2 | 23496775 | 7 | 0.02 | 98.15 | 94.57 | 51.58 |
| A26_CK3 | FRAS210153716-9r | 22357935 | 6.7 | 20373167 | 6.1 | 0.02 | 98.25 | 94.7 | 52.7 |
| A26_CK4 | FRAS210153716-8r | 22927226 | 6.9 | 20910761 | 6.3 | 0.02 | 98.23 | 94.73 | 49.86 |
| B30_CK1 | FRAS210153717-6r | 22379191 | 6.7 | 21760183 | 6.5 | 0.03 | 97.98 | 93.87 | 47.5 |
| B30_CK2 | FRAS210153717-7r | 22911974 | 6.9 | 21140123 | 6.3 | 0.02 | 98.05 | 94.17 | 49.33 |
| B30_CK3 | FRAS210153717-8r | 23042918 | 6.9 | 22507731 | 6.8 | 0.03 | 97.97 | 93.82 | 46.94 |
| B30_CK4 | FRAS210153717-9r | 25577846 | 7.7 | 24176801 | 7.3 | 0.02 | 98.09 | 94.21 | 47.94 |

**Table S-6.** Mapping rate of total reads in each sample

| Sample name | Total reads | Total mapped | Percentage |
| --- | --- | --- | --- |
| CK1 | 44062460 | 39064166 | (88.66%) |
| CK2 | 42990710 | 37953976 | (88.28%) |
| CK3 | 40824310 | 36526548 | (89.47%) |
| CK4 | 42088402 | 37461126 | (89.01%) |
| A26_CK1 | 46454516 | 41239634 | (88.77%) |
| A26_CK2 | 46993550 | 38797790 | (82.56%) |
| A26_CK3 | 40746334 | 34409176 | (84.45%) |
| A26_CK4 | 41821522 | 36809768 | (88.02%) |
| B30_CK1 | 43520366 | 38881460 | (89.34%) |
| B30_CK2 | 42280246 | 35093664 | (83.00%) |
| B30_CK3 | 45015462 | 40652934 | (90.31%) |
| B30_CK4 | 48353602 | 42410692 | (87.71%) |

**Table S-7.** Top 20 enriched metabolic pathways identified through KEGG enrichment analysis

| Sr. No | Pathway annotation | Number of metabolites | P-value | Rich Factor | -log(p-value) |
| --- | --- | --- | --- | --- | --- |
| 1. | Tyrosine metabolism | 23 | 1.3314E-08 | 0.294871795 | 7.875692327 |
| 2. | Phenylalanine metabolism | 16 | 1.01645E-05 | 0.266666667 | 4.992915453 |
| 3. | Valine, leucine, and isoleucine biosynthesis | 9 | 3.41111E-05 | 0.391304348 | 4.467103718 |
| 4. | Pyrimidine metabolism | 14 | 0.000440084 | 0.215384615 | 3.356464222 |
| 5. | ABC transporters | 12 | 0.063053936 | 0.129032258 | 1.200287796 |
| 6. | Glycine, serine, and threonine metabolism | 8 | 0.041684592 | 0.16 | 1.380024442 |
| 7. | Galactose metabolism | 11 | 0.000715572 | 0.239130435 | 3.14534686 |
| 8. | Vitamin digestion and absorption | 7 | 0.024372381 | 0.189189189 | 1.613102042 |
| 9. | beta-Alanine metabolism | 6 | 0.037619304 | 0.1875 | 1.424589238 |
| 10. | cAMP signaling pathway | 7 | 0.002590384 | 0.28 | 2.586635798 |
| 11. | Tryptophan metabolism | 14 | 0.005174888 | 0.168674699 | 2.286099079 |
| 12. | Linoleic acid metabolism | 7 | 0.005187943 | 0.25 | 2.285004788 |
| 13. | Alanine, aspartate and glutamate metabolism | 4 | 0.005630925 | 0.4 | 2.249420253 |
| 14. | Prolactin signaling pathway | 4 | 0.008301334 | 0.363636364 | 2.080852094 |
| 15. | Citrate cycle (TCA cycle) | 4 | 0.06921756 | 0.2 | 1.159783714 |
| 16. | Aminoacyl-tRNA biosynthesis | 7 | 0.115750374 | 0.134615385 | 0.936477598 |
| 17. | Lysine degradation | 9 | 0.015507693 | 0.18 | 1.809452804 |
| 18. | Phenylalanine, tyrosine, and tryptophan biosynthesis | 7 | 0.015622223 | 0.205882353 | 1.806257154 |
| 19. | Purine metabolism | 14 | 0.016842331 | 0.147368421 | 1.773597812 |
| 20. | Protein digestion and absorption | 6 | 0.024081978 | 0.206896552 | 1.618307838 |
